# Emergency Response, Influence and Lessons in the 2021 Compound Disaster in Henan Province of China

**DOI:** 10.3390/ijerph19010488

**Published:** 2022-01-02

**Authors:** Zhengru Tao, Lu Han

**Affiliations:** Key Laboratory of Earthquake Engineering and Engineering Vibration, Institute of Engineering Mechanics, China Earthquake Administration, Harbin 150080, China; 13045107179@163.com

**Keywords:** compound disaster, rainstorm, pandemic, COVID-19, emergency response

## Abstract

Henan province, located in central China, suffered a heavy rainstorm and an outbreak of COVID-19 from the middle of July to the middle of August. We review and investigate the emergency response to these two events. The influence of the compound disaster on provincial economic operations, fixed assets, consumer goods, the logistics industry, high-tech manufacturing, and strategic emerging industries is analyzed in detail. Since the province’s economic situation has been positive for a long time, the influence of the compound disaster was short-term. The countermeasures to the pandemic were efficient since they had previously been in practice at various times in 2020. However, in the face of unusual disasters such as the rainstorm, the gap between early warning and emergency response needs to be bridged, and the sources of relief funds should be diversified.

## 1. Introduction

Henan is a central province of China and an important agricultural region, a comprehensive transportation hinge, and a communication hub. According to the official website of the province [1], its total area is 167,000 km^2^, 48.8% of which is farmland, and the terrain is high in the west and low in the east. Plain basins and mountain hills account for 55.7% and 44.3% of the total area, respectively. It has a continental monsoon climate ranging from the northern subtropical climate to a warm temperate zone, with four distinct seasons and frequent climate disasters. In the last decade, the average annual temperature was 12.9 °C to 16.5 °C, and the average annual precipitation was 464.2–1193.2 mm. It is one of China’s most populous provinces, with a total population of 109.52 million and a permanent population of 96.4 million at the end of 2019. In 2020, the province’s GDP was CNY 5499.707 billion, and total grain output reached a record of 68.25 billion kilograms. The total fiscal revenue was CNY 626.739 billion, of which the general public budget revenue reached CNY 415.522 billion, and the per capita disposable income of residents was CNY 24,810.1. The province administers 39 cities, 83 counties, 53 municipal districts, 1791 towns, and 662 sub-district offices, and the capital is Zhengzhou.

In the middle of July 2021, the province was hit by unusually heavy rainfall, which triggered severe floods. The event was named the 7.20 Henan rainstorm. It burst the banks of rivers and overwhelmed dams, causing severe waterlogging, traffic disruption, and power outages, and upending the lives of tens of millions. According to the 10th press conference on “Henan Province Flood Control and Disaster Relief” held by the Information Office of the provincial government on August 3 [2], as of 12:00 on August 2, the direct economic loss was CNY 114.269 billion, 302 people had died, and 50 people were missing. A total of CNY 8.271 billion of emergency disaster relief funds were allocated. At the end of July, the focus was shifted from emergency rescue and relief to recovery and reconstruction, while post-disaster pandemic prevention was emphasized by local authorities. However, on July 31, 12 local confirmed cases and 20 local asymptomatic cases of COVID-19 appeared in Zhengzhou [3]. At the press conference held by the joint prevention and control mechanism of the State Council on August 5 [4], this was classified as a local outbreak of the pandemic caused by a nosocomial infection that was highly homologous with the gene sequencing of an overseas imported case treated in a designated hospital; it was also a Delta mutant strain. Most of the infected people were associated with the hospital. In this study, we investigated the emergency response to the 7.20 Henan rainstorm compounded with the outbreak of COVID-19, together with the effects at the provincial level, drawing several lessons.

## 2. Materials and Methods

### 2.1. Emergency Response to the 7.20 Henan Rainstorm

#### 2.1.1. July 20 Henan Rainstorm

**Figure 1:**
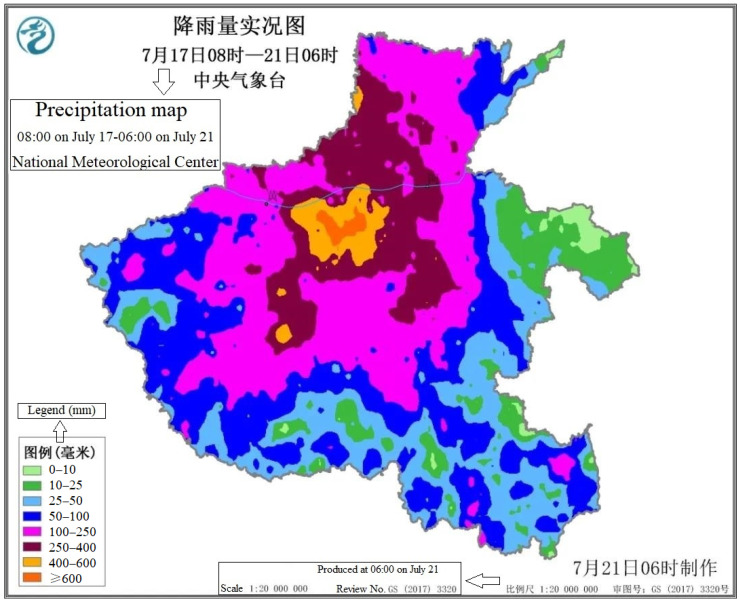
The precipitation map from 8:00 on July 17 to 6:00 on July 21 (redrawn from [7]).

The heavy rainfall across Henan began on July 16; a number of large and medium-sized reservoirs exceeded the normal limits, and floods occurred in some mountainous areas. The Provincial Flood Control and Drought Relief Headquarters issued commander’s order No. 1 in 2021 [5], requiring efforts to be made to prepare for future heavy rainfall disasters and effectively protect people’s lives and property; in case of dangerous situations, threatened people should be rescued and transferred quickly, in order to avoid mass deaths and injuries. According to the Provincial Flood Control Emergency Press Conference [6], from 8:00 on July 17 to 14:00 on July 21, a rare weather event of continuous heavy rainfall occurred in the province, with heavy rain in all cities of the province. During this heavy rainfall event, the average precipitation over the province was 150.5 mm, the maximum average city-level precipitation was 461.7 mm in Zhengzhou, and hourly precipitation of 201.9 mm appeared in Zhengzhou from 16:00 to 17:00 on July 20, exceeding the historical extreme value on the Chinese mainland. Ten national meteorological stations had broken the historical record since the establishment of the stations. The precipitation map, from 8:00 on July 17 to 6:00 on July 21, is shown in Figure 1, as provided by the National Meteorological Center [7].

The Provincial Meteorological Department issued 11 highest-level warnings for the rainstorm, at 21:50 and 21:59 on July 19, at 05:50, 09:00, 11:50, 12:55, 16:20, 19:20, and 21:40 on July 20, and at 00:40 and 03:40 on July 21 [8]. A highest-level warning means that the rainfall will continue for the next 3 h, and the cumulative precipitation will reach more than 100 mm. From 00:00 on July 20 to 00:00 on July 21, the average precipitation reached 302 mm, and a 24 h maximum precipitation of 702.0 mm was recorded in Houzhai, Erqi District of Zhengzhou, from 03:00 on July 20 to 03:00 on July 21, exceeding the average annual precipitation in Zhengzhou.

#### 2.1.2. Emergency Response

The Provincial Flood Control and Drought Relief Headquarters launched an emergency response to flood control at level IV at 17:00 on July 19, which was upgraded to level II at 18:00 on July 20 and upgraded to level I at 3:00 on July 21.

**Figure 2:**
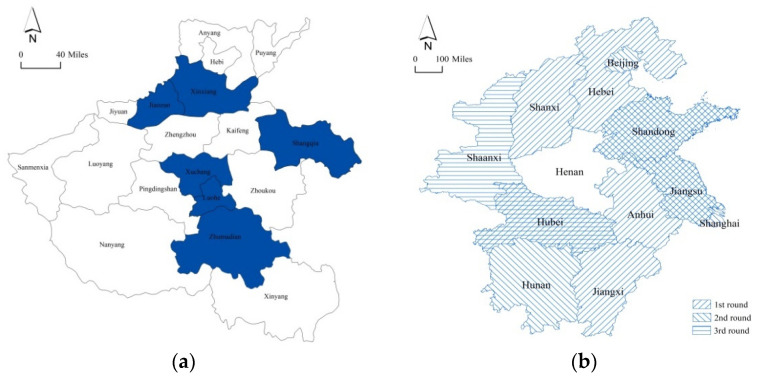
The location of reinforcement provinces/cities: (**a**) reinforcement from six cities in Henan province; (**b**) three rounds of reinforcements from other provinces/cities.

On the China Emergency Information [9], the Fire and Rescue Department of Henan Province raised the rescue level on July 20 and urgently dispatched rescue forces from Jiaozuo, Xuchang, Luohe, Xinxiang, Shangqiu, and Zhumadian, as shown in blue in Figure 2a, including 180 firefighters, 43 fire engines, and 22 boats, to help Zhengzhou to carry out personnel search and rescue, drainage, and transfer of people. On July 21, the Ministry of Emergency Management of the People’s Republic of China initiated cross-regional reinforcements of the fire and rescue teams, as shown as Figure 2b. In the first round, 7 professional provincial fire and rescue teams from Hebei, Shanxi, Jiangsu, Anhui, Jiangxi, Shandong, and Hubei were dispatched overnight, including 1800 firefighters, 250 boats, 7 sets of high-power drainage vehicles, 11 sets of remote water supply systems and more than 18,500 items of flood rescue equipment. At 22:00 on July 22, the second round of cross-regional reinforcements was launched, and 510 firefighters, 64 remote water supply and drainage vehicles, and 100 rubber boats were dispatched from the 5 provinces/cities of Beijing, Shanghai, Jiangsu, Shandong and Hunan, to carry out drainage rescue and relief overnight. On the night of July 23, the third round was launched, including two emergency drainage teams of 300 members from Hubei and Shaanxi provinces and 103 sets of drainage equipment, sent to the city of Xinxiang to carry out drainage rescue. As of August 2, 4859 firefighters, 532 boats, and 256 sets of large-scale drainage equipment were rushed to Henan; more than two million people participated in the rescue. On August 4, the cross-regional rescue forces left Henan after completing the relief task and implementing the normal nucleic acid tests and pandemic prevention measures in accordance with the local pandemic prevention policies [10].

Figure 2b shows that the cross-regional forces were sent from the southern neighboring provinces to the more distant and more western provinces/cities; however, in Figure 2a, the rescue forces were from several farther cities.

On July 21, 3.356 million kilowatts of power was supplied by the Henan power grid, reduced by 2.224 million kilowatts compared with July 20, affecting 3.7433 million users, and 1.484 million kilowatts was supplied by the Zhengzhou power grid, reduced by 1.844 million kilowatts compared with July 20. The load loss was 1.3 million kilowatts, affecting 749,600 users [6,11]. Nearly 20,000 technicians from electric power companies, mobilizing 462 power generators and more than 1000 other generators, were dispatched from 24 provinces to Henan. As of August 8, the 42 power substations, 47 transmission lines, and 98.5% of the 1807 damaged distribution lines had been restored, 98.3% of the 58,000 distribution stations had been recovered, and 98.5% of the non-serving communication base stations had been reinstalled [12].

**Figure 3:**
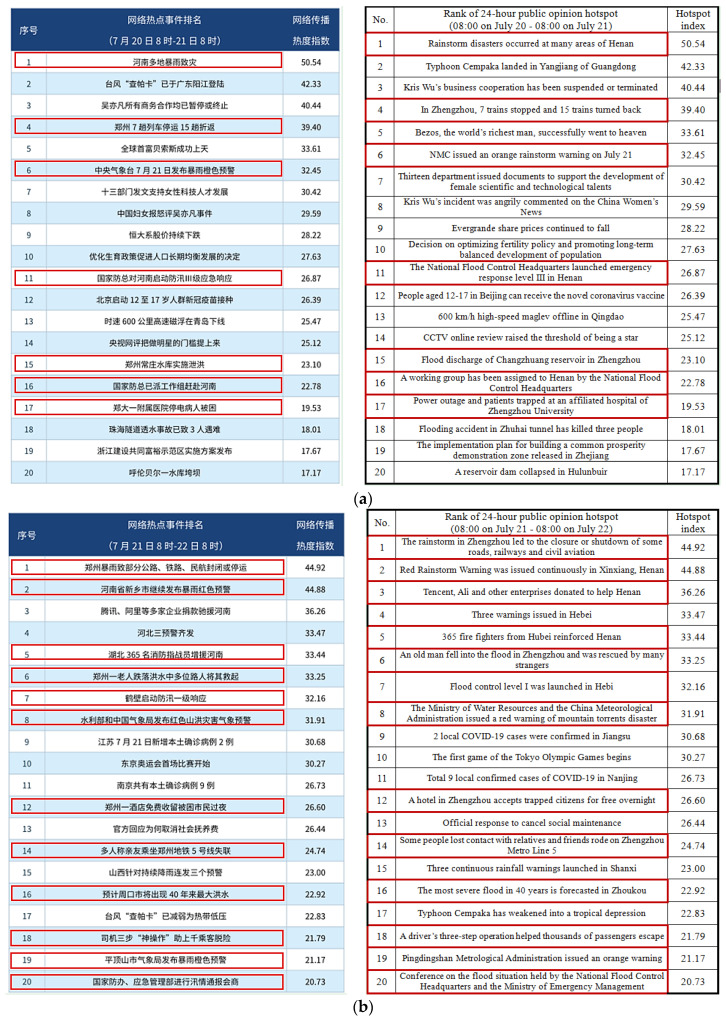
Public opinion hotspots from Sina Public Opinion: (**a**) hotspots on July 21; (**b**) hotspots on July 22 (redrawn from [11]).

On July 21 and July 22, the rainstorm was at the top of the hotspot event list, according to the report of the 24 h network public opinion hotspots from Sina Public Opinion (08:00 on July 20–08:00 on July 21 and 08:00 on July 21–08:00 on July 22) [13], as shown in Figure 3a,b, in which 7 out of 20 hotspots and 13 out of 20 hotspots concerned this event (those in red rectangles).

**Figure 4:**
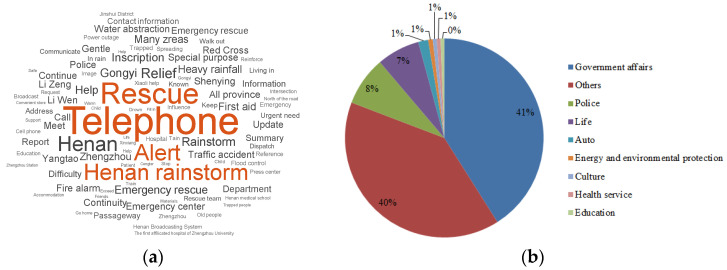
Hotspots regarding the Henan rainstorm from Sina Public Opinion: (**a**) keyword cloud; (**b**) proportion of information by industry.

From a search on the words “Henan rainstorm” in the period from July 9 to July 28 on Sina Public Opinion [13], which investigates public opinion on network media such as Weibo, websites, WeChat, clients, digital newspapers, and videos, a keyword cloud was obtained, as shown in Figure 4a. Here, 9 out of the top 10 keywords concerned rescue and relief in the rainstorm, including telephone, rescue, alert, Henan rainstorm, Henan, relief, rainstorm, Gongyi, and help. The information is classified by industry, and the proportions are shown in Figure 4b, indicating that information from government administrations was disclosed promptly during the process of emergency rescue.

**Figure 5:**
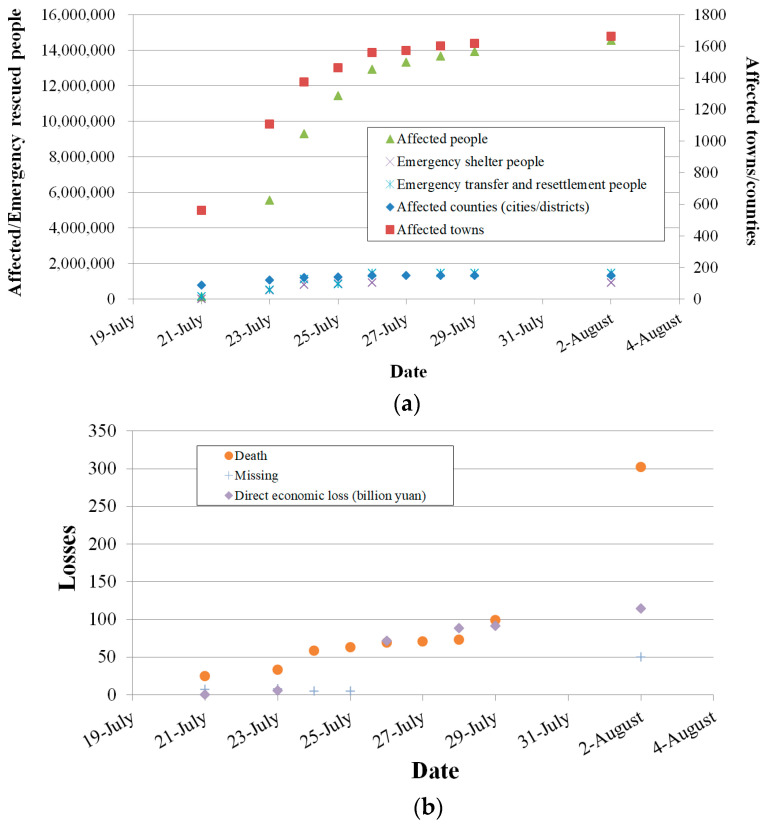
Data on losses and affected and rescued people: (**a**) people affected and rescued; (**b**) losses.

The information office of the provincial government held 10 press conferences on flood control in Henan province to release information on rainfall and relief, from July 21 to August 9. Data released at the conferences on casualties, direct losses, affected areas and people, and emergency rescues are shown in Figure 5.

As Figure 5 shows, the county-level situation was known in 5 days, and information on affected towns and people was known in 8 days. This means that more time was spent on gathering detailed information, due to the failure of electricity supplies. The processes of providing emergency shelter, transfer and resettlement were consistent with the situation at the county level. The precise number of casualties was given up to August 2, with a total of 302 deaths. Of the 50 people missing, 47 were in Zhengzhou and 3 were in Xinxiang. As of August 9, 150 counties (cities, districts), 1664 towns, and 14.814 million people were affected; 933,800 people were organized for emergency transfer and resettlement, and among these the number of centrally resettled people dropped from 584,200 to 193, and the resettlement places dropped from 2126 to 5. The affected area of crops was 10,802 km^2^, the disaster area was 6673.3 km^2^, and the dead harvest area was 3424.7 km^2^. A total of 35,325 houses collapsed and 53,535 houses were heavily damaged. The direct loss was evaluated as CNY 133.715 billion, with a total of CNY 9.031 billion yuan allocated for disaster relief.

### 2.2. Emergency Response to COVID-19

#### 2.2.1. The Outbreak of COVID-19

At the time of the rainstorm, pandemic prevention, recovery, and reconstruction were promoted. A total of 15 medical teams went to the 15 worst-hit counties, cities, and districts to strengthen monitoring, disinfection of drinking water, and food hygiene. A total of 1824 km^2^ were sterilized, and on August 9, this reached 3170 km^2^. All the centralized resettlement areas, public transport, and flooded places were covered, and work and production resumption reached 94.6%. The pandemic prevention measures protected a large number of people from COVID-19 during the provision of shelter, transfer, and resettlement.

**Figure 6:**
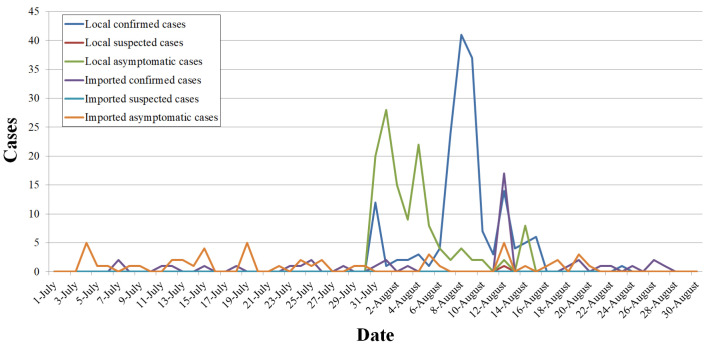
Local and imported cases of COVID-19.

According to the Health Commission of Henan Province, on July 31, 12 local confirmed cases appeared suddenly in the province. No new confirmed cases had been reported for 124 days since March 29. The local and imported cases recorded over the following month are shown in Figure 6.

**Figure 7:**
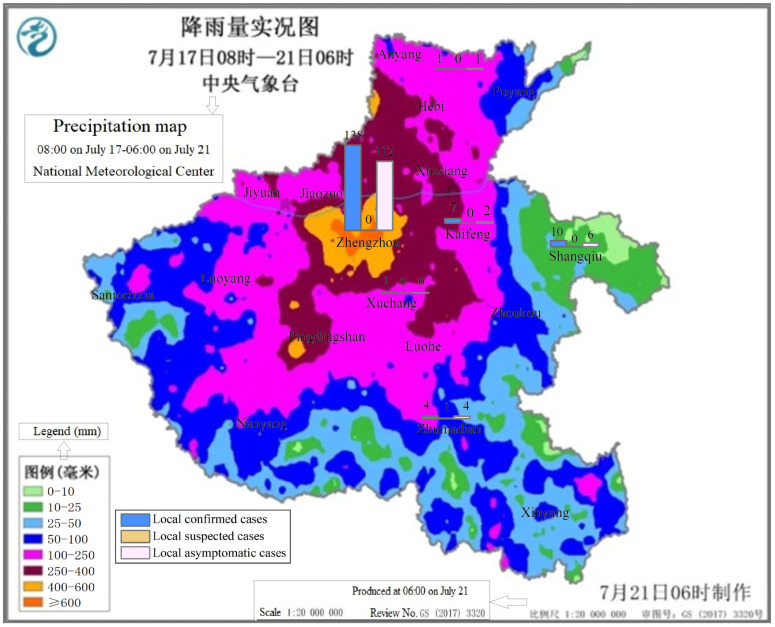
Distribution of local pandemic cases overlaid on the precipitation map.

The daily local asymptomatic cases reached a peak of 28 on August 1, and the local confirmed cases reached 41 on August 8. Up to October 15, no new confirmed cases had been reported for 53 days since August 24. From July 31 to August 24, there were 167 local confirmed cases in this province. The distribution of local confirmed cases, local suspected cases, and local asymptomatic cases in each city is overlaid on Figure 1, as Figure 7.

Figure 7 shows that there was no relation between the precipitation and the number of local cases, which means there was no influence from the rainstorm on the spread of disease; however, the capital of the province was affected by both.

#### 2.2.2. Emergency Response

The first positive case was found by the initial screening of nucleic acid during routine examination in the Erqi District of Zhengzhou at 17:00 on July 30, and the community was locked down and the epidemiological investigation expanded that night [14]. From 10:00 on August 2, “personal nucleic acid certificate inspection service points” were set up at all expressway stations and national and provincial roads in Zhengzhou, to check that drivers and passengers had negative nucleic acid test certificates issued within 48 h [15]. The lockdown areas were adjusted continuously according to the distribution of confirmed and asymptomatic cases, and all residents had nucleic acid tests on the 1st, 4th and 7th days of lockdown [16].

As of 16:00 on August 4, the sampling work for the first round of nucleic acid tests for all residents in Zhengzhou (11.18 million people) was completed [16]. The sampling work for the second round took place from 9:00 on August 5 to 17:00 on August 6 [17], and the testing of 10.8329 million residents was completed as of 17:00 on August 7 [18]. The sampling work for the third round was from 15:00 on August 8 to 24:00 on August 9, and the testing of 11.9517 million residents was completed as of 24:00 on August 10 [19]. The sampling work for the fourth round was from 9:00 on August 15 to 22:00 on August 17, and the testing of 11.8896 million residents was completed. A total of 15.1691 million doses of the novel coronavirus vaccine were received, as of 24:00 on August 16 [20].

According to the second press conference on COVID-19 prevention and control, held by the Zhengzhou COVID-19 Leading Group Office [21], after genetic sequencing, the first two infected persons were found to be highly homologous with the confirmed patients who entered from Myanmar and were treated in the Sixth Municipal Hospital, and all strains identified were Delta variant strains. Most of the infected people were associated with the Sixth Municipal Hospital. Since the rules and regulations relating to pandemic prevention and control were not implemented by the local health administration and the hospital, the local government has held relevant responsible persons seriously accountable.

## 3. Influence of the Compound Disaster

### 3.1. Influence on Provincial Economic Operations

In the reports on the province’s economic operation in July and August, released by Henan Province Bureau of Statistics [22,23], the economic operation encountered unprecedented severe challenges.

Agricultural production suffered a certain impact, but replanting work was carried out efficiently. The affected area of crops accounted for about 13.6% of the total sowing area in autumn, with about 4.3% wiped out completely. Where conditions permitted, mung beans, vegetables, and other crops with a short growth period were replanted.

**Figure 8:**
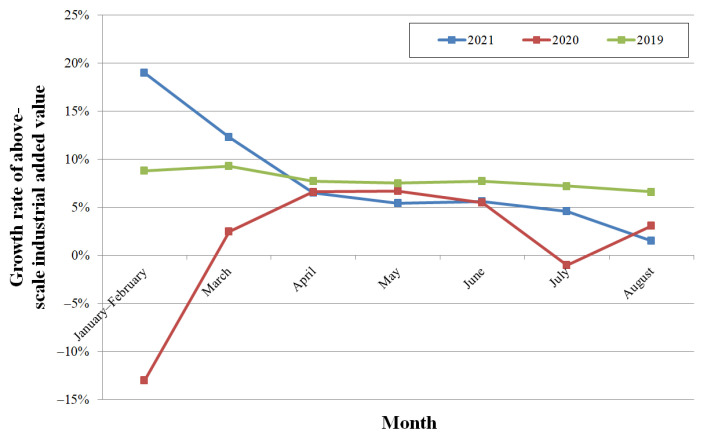
The YoY growth rate of provincial above-scale industrial added value.

Industrial production declined. Provincial above-scale industrial added value increased year-on-year (YoY) by 4.6% in July, which was 1% down from the previous month, and increased by 1.5% in August, which was 3.1% down from July. As shown in Figure 8, the influence of COVID-19 was significant during the first quarter of 2020, and the influence of the compound disaster appears in August 2021. Compared with the YoY growth rates in the last two years, from April, these were lower than those in 2019 and close to those in four months of 2020.

Traditional industries, mainly labor-intensive industries, which accounted for 48.9% of provincial above-scale industrial added value, declined significantly, as negative growth was observed for the first time in 2021, with a decrease YoY of 0.4% in July, which was 3.2% down from June. This caused the growth rate of the provincial above-scale industrial added value to decline by 0.2%. However, the consumer goods manufacturing industry recovered quickly in August, and its added value increased by 7.8%.

Affected by the rainstorm, some sections of the transportation system were overwhelmed and even collapsed, which led to the cargo turnover increasing YoY by 9.9% in July, which was 1.2% down from June. The railway freight turnover fell YoY by 12.6%, which was 17.4% down from the previous month.

### 3.2. Influence on Fixed-Asset Market

**Figure 9:**
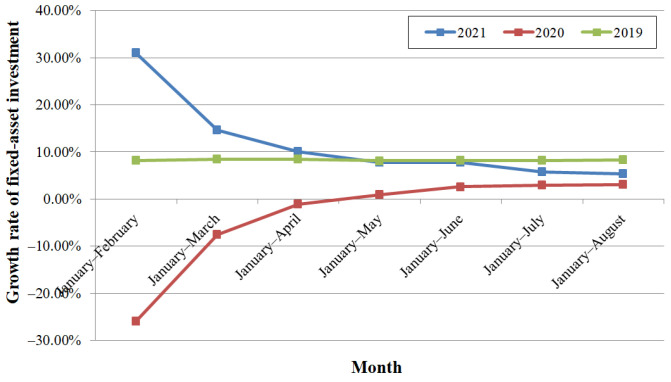
The YoY growth rate of fixed-asset investment.

Investment growth in fixed assets, infrastructure, and real estate decreased. The provincial fixed-asset investment from January to July increased by 5.8% YoY, which was 2.0% down from the first half year, and increased YoY by 5.4% from January to August, which was 0.4% down from the period between January and July. However, the gap between the provincial level and the national level was narrowed, which indicates that the national fixed-asset investment had decreased. Comparing the growth rates in last two years, as shown in Figure 9, the influence of COVID-19 was clearly significant during the first half of 2020, and the influence of the compound disaster was significant in July and August of 2021. From January to April, the growth rates in 2021 were higher, indicating recovery from the pandemic in 2020. Those from January to May and from January to June were close to those in 2019 and higher than those in 2020; those from January to July and from January to August were lower than those in 2019 and higher than those in 2020. From January to July, investment in infrastructure increased by 3.6% YoY, which was 6.0% down from the first half year, and the growth rate was lower than that of total investment for the first time in 2021. From January to August, the growth rate increased by 1.7%.

**Figure 10:**
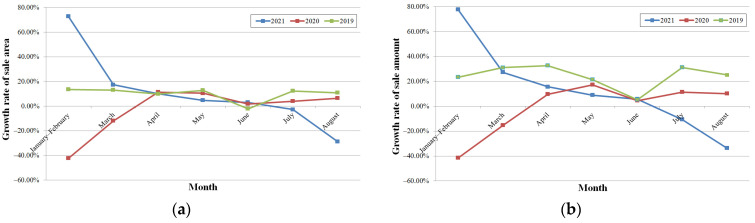
The YoY growth rate of commodity housing sales (data from Henan Province Bureau of Statistics): (**a**) the YoY growth rate of sale area; (**b**) the YoY growth rate of sale amount.

The growth rates of sale areas of commodity housing in July and August fell by 2.8% and 28.9%, and those of sale amounts declined by 10.68% and 33.79%, respectively, as shown in Figure 10. The influence of COVID-19 was significant during the first four months of 2020, and the influence of the compound disaster was more significant in August of this year. Compared with the growth rates in 2019, those of sale areas were similar from March to June, and those of sale amounts were lower in April and May; the growth rates of sale areas were higher than those of sale amounts, which indicates that the price was lower in 2021.

### 3.3. Influence on Consumer Goods Market

**Figure 11:**
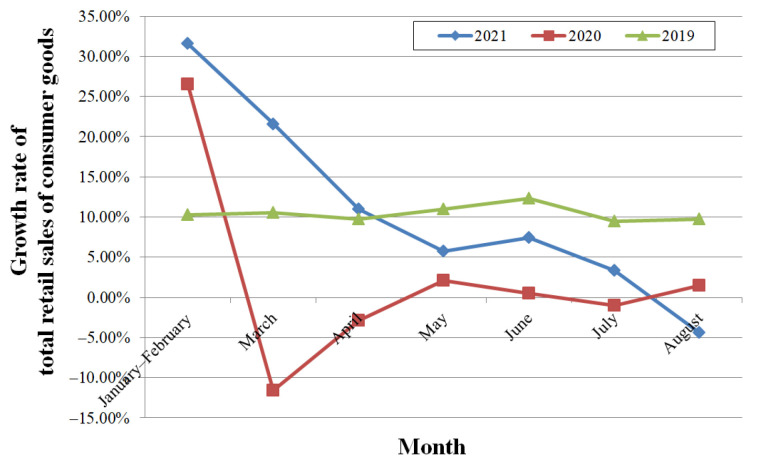
The YoY growth rate of total retail sales of consumer goods.

The growth rate of total retail sales of consumer goods increased YoY by 3.3% in July, which was 4.1% down from June, and declined by 4.4% in August, as shown in Figure 11. The influence of COVID-19 was significant during the first four months of 2020, and the influence of the compound disaster was more significant in August of this year. Compared with the growth rates in last two years, from May, the rates were lower than those in 2019 and lower in August than in 2020.

**Figure 12:**
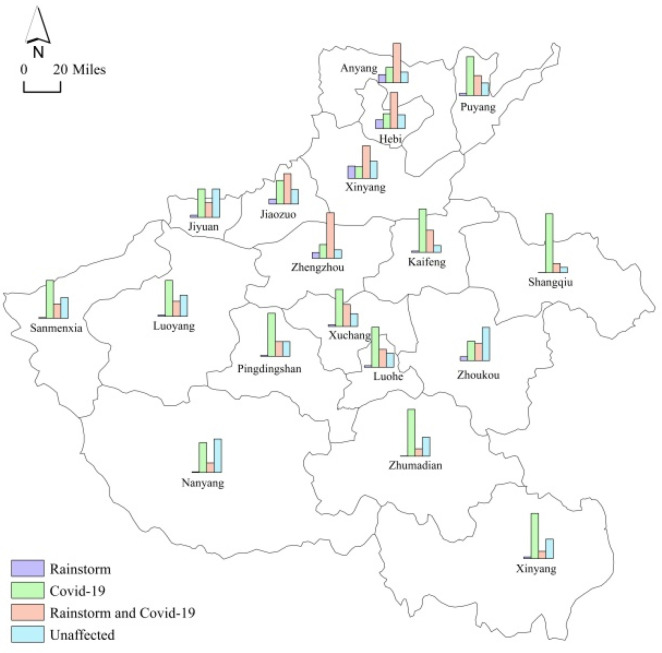
Proportion of affected and unaffected enterprises in each city of Henan province.

To understand the impact of the compound disaster on the provincial consumer goods market, Henan Province Bureau of Statistics carried out a questionnaire survey of 15,340 wholesale and retail, accommodation, and catering enterprises, with a submission rate of 82.8%, from August 18 to August 23 [24]. Of the 12,709 enterprises that filled in the questionnaire, 4.2%, 40.8%, and 30.4% were affected by the rainstorm, COVID-19, or both, respectively; 73.3% of wholesale and retail enterprises and 85.4% of accommodation and catering enterprises were affected. The impact on cities was quite different: the proportion of enterprises affected by the rainstorm, the pandemic, or both, and the proportion of unaffected enterprises in each city of Henan province are shown in Figure 12.

As shown in Figure 12, the effect on enterprises in Zhengzhou and the four cities to the north was deepened by the compound disaster, which is consistent with the high-precipitation areas in Figure 1. Enterprises in other cities were affected mostly by the pandemic.

According to the resumed production conditions reported by 10,759 enterprises, 53.7% enterprises recovered to over 70% of the normal production level, comprising 56.2% of wholesale and retail enterprises and 38.6% of accommodation and catering enterprises. The main difficulties in production and operation caused by the compound disaster were operating costs rises, pandemic prevention and control policies, insufficient new orders, and problems with warehousing and delivery transportation. Wholesale and retail enterprises were mainly affected by operating costs rises; accommodation and catering enterprises were mainly impacted by pandemic prevention and control policies.

**Figure 13:**
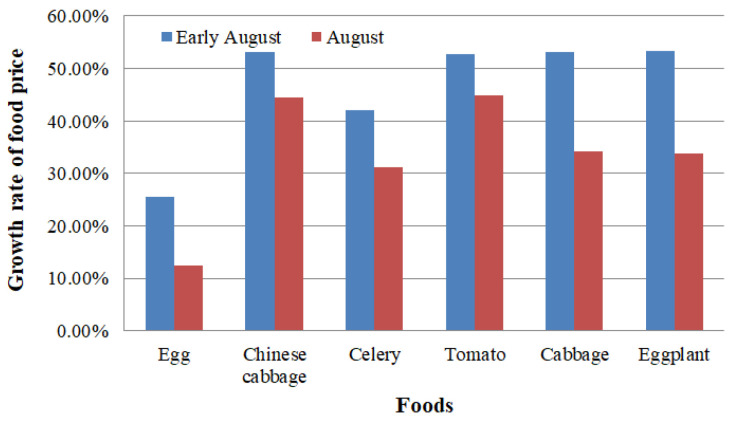
The YoY growth rate of some typical foods.

Due to the sufficient supply in major food markets, most prices were stable, but the prices of eggs and 13 types of vegetables rose significantly. The YoY growth rates of these food prices are shown in Figure 13, according to the monitoring results of Henan Province Bureau of Statistics [25,26]. In early August, production dropped, roads were damaged, traffic flow was controlled, and the costs of labor and transportation increased. These factors caused the price of some foods to rise. There was a gradual recovery in production and living costs after the rainstorm, and all major food prices showed a downward trend from mid-August onwards.

**Figure 14:**
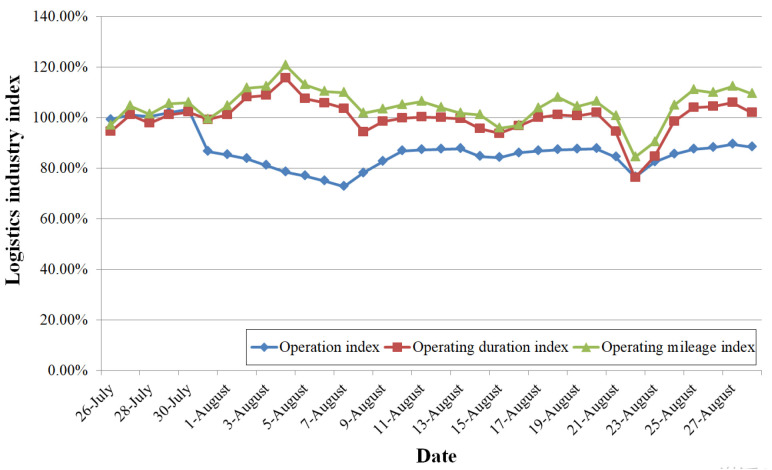
Logistics industry index during the pandemic.

Cold chain material distribution and the logistics industry were strongly affected. The logistics industry indices during the pandemic according to the report on the resumed production index of the logistics industry published by Henan Development and Reform Commission, are shown in Figure 14.

By July 30, the negative influence of the rainstorm on the operational indices of logistics vehicles had been eliminated. From July 31, the number of operating vehicles (operation index) declined, reaching 74.98% of normal levels on August 6. Meanwhile, the operating duration and mileage increased, indicating that the vehicles operated at high load. This situation continued until August 27; although the operating duration and mileage returned to the normal level, the operation index was lower than 90%. Logistics operation speed slowed down significantly, some lines were suspended, and the efficiency was reduced, due to the pandemic prevention policies.

A positive impact of the compound disaster was seen in high-tech manufacturing and the strategic emerging industries, which maintained rapid growth. The added values of the high-tech manufacturing industry and strategic emerging industries increased by 36.3% and 13.7% in July, which was higher than the growth rate of the above-scale industrial added value by 31.7% and 9.1%, respectively. Those in August increased by 19.3% and 8.5%, which was higher than the growth rate of the above-scale industrial added value by 17.8% and 7.0%. Investment in the high-tech manufacturing industry also increased rapidly. From January to July, it increased by 32.2% YoY, which was higher than the growth rate of industrial investment by 22.9%. From January to August, it increased YoY by 38.1%.

In general, due to the great losses from the compound disaster, economic operation was affected to varying degrees; however, the long-term positive trend of the provincial economy had not been shaken. Thus, the influence was short-term, albeit with large uncertainty.

## 4. Discussion of Some Lessons

### 4.1. Lack of Strict and Practical Measures at the Highest Warning Level

According to the 10th press conference on flood control in Henan province on August 2, the total number of deaths was 302, of which 292 were in Zhengzhou, 7 in Xinxiang, 2 in Pingdingshan, and 1 in Luohe. Of the 292 deaths in Zhengzhou, 189 deaths were caused by floods and mudslides, 54 were caused by collapsed houses, and 39 were caused by drowning in basements, garages, underground pipe galleries, and other underground spaces. Of the 39 deaths caused by deep water in underground spaces, 14 were along Zhengzhou Metro Line 5 and 6 were in the Jingguang North Road Tunnel, on July 20, which attracted public attention. We analyze these two cases in detail to learn lessons on emergency response in central urban areas.

**Figure 15:**
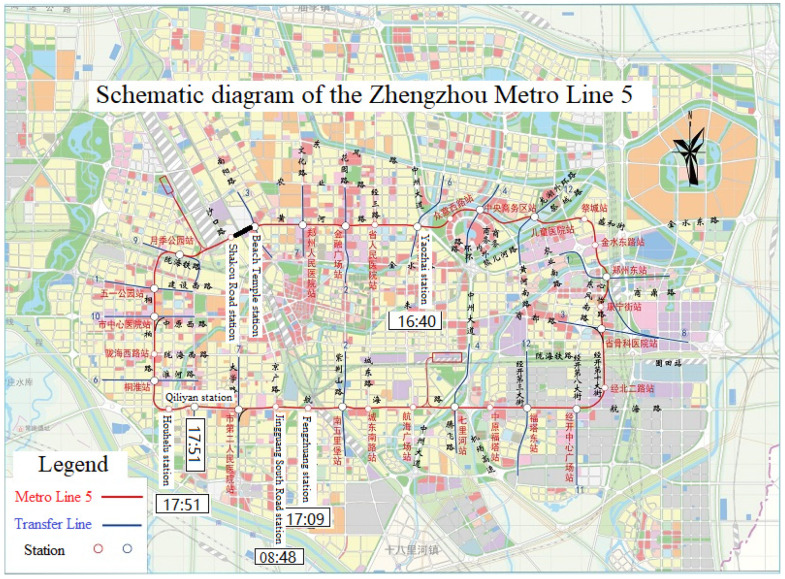
Schematic diagram of the Zhengzhou Metro Line 5 (redrawn from [27]).

Zhengzhou Metro Line 5 was constructed on 18 December 2014 and ran an air-load trial on 29 December 2018. As of 14 May 2021, the total length of the line was 40.7 km, and it had a total of 32 stations, all underground. This is the only circular line of the existing Metro lines, as shown in Figure 15. Connections to the downtown area and the surrounding areas are made by transferring to other lines.

**Figure 16:**
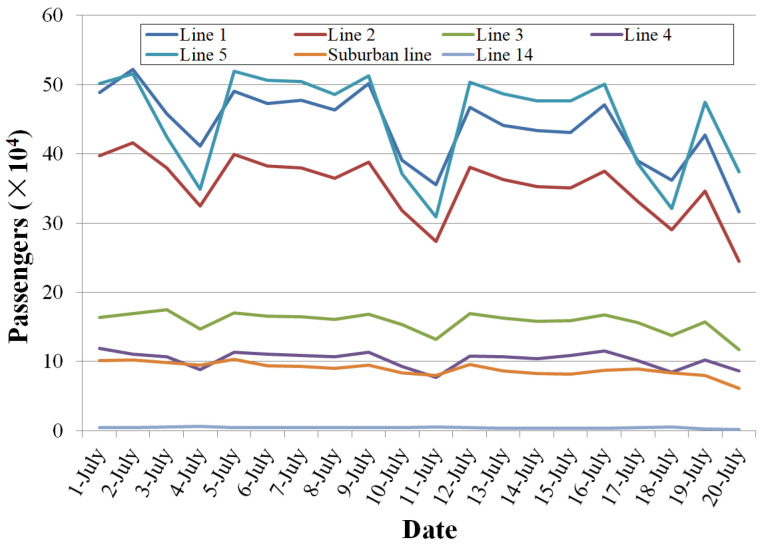
Daily passengers for seven Metro lines.

It has the largest daily passenger capacity of the seven existing Metro lines on weekdays, according to the data from the Weibo of Zhengzhou Metro [28], as shown in Figure 16.

On July 20, serious ground waterlogging occurred around the Wulongkou parking area on the line, caused by heavy rainfall. At about 18:00 in the rush hour, water gushed across the retaining wall of the entry and into the main line area, which resulted in a train stopping between the Shakou Road station and the Beach Temple station, shown as a black line in Figure 15. More than 500 people were stranded [29]. On the same day, some entries to other stations on the line were closed successively; the times are also labeled in Figure 15. At 17:00, the emergency response level in Zhengzhou was upgraded to the highest level. At 18:42, on the Weibo of Zhengzhou Metro, it was announced that the whole Metro system was closing down. Line 5 was out of service until September 14.

**Figure 17:**
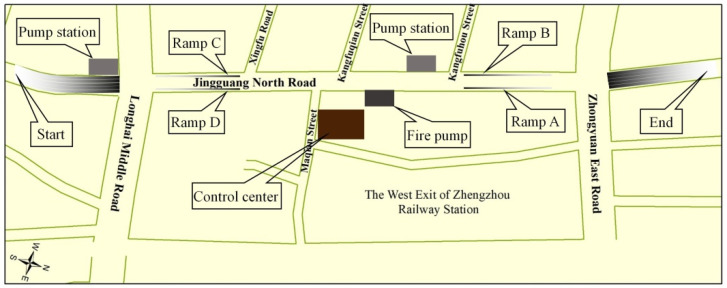
The floor plan of Jingguang North Road Tunnel (redrawn from [31]).

Jingguang North Road Tunnel is a part of the Jingguang Expressway system, which is in the south section of the north–south expressway. Construction was started on 17 December 2009, and it opened to traffic on 28 April 2012. The total length of the tunnel is 1.8 km, composed of a buried section of 1.36 km and an open section of 475 m, and the maximum height difference between the ground and the tunnel plate is about 9 m. The depth of ground water is 11.8–15.7 m, and the historical highest level was at a depth of about 10.0 m. This tunnel is around 300 m away from the west square of the Zhengzhou Railway Station, and contributes to distributing the passing passengers quickly. The drainage system in the tunnel is designed to drain away the water from the open section in the rainy season, the ground water brought into the tunnel by vehicles, the daily flushing wastewater, fire wastewater, and a little leakage of water. This system is composed of three parts: a reverse slope is set at the entrance to block the ground water in the rainy season. a rectangular drainage ditch is set on the right side of the lane, and two underground pump stations are set to the west of the tunnel, as shown in Figure 17. With a design return period of 50 years and a flow capacity of 0.52 m^3^/s and 0.5 m^3^/s, respectively, there are three pumps at each station, which are triggered automatically by a certain water depth [30,31].

The other drainage system is designed to drain away the ground water from an area of about 2.78 km^2^ along the Jingguang Road, draining it into Jinshui River and Xionger River with a flow capacity of 2.8 m^3^/s and 7.3 m^3^/s, respectively. Some of the main problems of flood control and drainage were discussed in detail a decade ago in [30]: (1) the long distance and large depth of the buried section were the hidden factors leading to stalled and flooded vehicles during the rainy season; (2) the area of 6.75 km^2^ around the tunnel was one of the most waterlogged areas in Zhengzhou, and when the standing water could not be discharged by the drainage systems, it would flow toward the lowest area; (3) the water levels of Jinshui River and Xionger River were high, which was not conducive to drainage; (4) the flow capacity of the ground drainage system was much higher than that of the two pump stations in the tunnel drainage system.

At around 16:00 on July 20, vehicles started to be blocked at the south exit of the tunnel, and ground water flowed from south to north into the tunnel half an hour later. At the 5th press conference on flood control in Henan Province on July 26, it was stated that the deepest water level was about 6 m, and 6 × 10^6^ m^3^ of water was discharged. A total of 247 vehicles were pulled out from the Jingguang Road Tunnel after it was drained by 30 high-power pumps operating over five days for 24 h/day.

From July 18, warnings of heavy rain were posted on “Zhengzhou Meteorology” which is the official Weibo of Zhengzhou Meteorology Bureau [32]. At 21:59 on July 19, 06:02, 09:08, 11:50, 16:01, and 21:32 on July 20 and 00:25 on July 21, the Zhengzhou Meteorology Bureau issued seven red warning alerts for a rainstorm, which was the highest warning level and meant that the precipitation in the following 3 h would amount to more than 100 mm.

According to [33], the designed lifetime of the main structures of the Metro was 100 years, and the drainage capacity was the rainwater amount in the 50-year recurrence period. In the “Zhengzhou City of Urban Rail Transit Operation and Management Measures”, released in 2013, it was stated that the operation unit could suspend the line operation or the operation of some road sections in the event of natural disasters and severe meteorological conditions that seriously affected the operational safety of the urban rail transit system if measures could not be taken to ensure safe operation, and that this should be announced to the public in good time.

According to Zhengzhou Meteorology, the hourly and daily precipitation in this rainstorm were likely to occur once in a thousand years, exceeding the designed drainage capacity of Metro Line 5 and the Jingguang North Road Tunnel. However, there are several lessons to be learnt. Firstly, the emergency response should be practicable. In the “Henan Province Meteorological Disaster Prevention Regulations”, released in 2009 and revised in 2020, it was stated that the meteorological stations affiliated to the competent meteorological authority are in charge of forecasting and warning of disastrous weather conditions, and they should report to the local government and the competent meteorological authority immediately and notify the relevant authorities. In the case of major meteorological disasters, the local governments and relevant authorities should, in accordance with the early warning level and the emergency plan for meteorological disasters, take the necessary emergency response measures such as shutdown, business suspension, class suspension, and traffic control in a timely fashion. There is a gap between the highest rainstorm warning issued by the competent agency and the emergency decisions made by the governors, which should be bridged in practice. Once the highest-level warning has been issued, the countermeasures should be executed strictly and immediately, rather than in the form of recommendations. For example, after the red warning signals issued on July 19 and early on July 20, notices regarding shutdown, business suspension, class suspension, and traffic control should have been issued to avoid people being exposed to the rainstorm. Secondly, an early warning system should be installed around areas that may potentially be waterlogged. In the rainy season, early warnings should be issued on the web, TV, broadcasts, and cell phones, and especially on the electronic screen on the road, or gates should be installed at intersections to stop vehicles from entering or to shut down Metro operation, based on the waterlogging situation at the lowest point, the forecasted precipitation, and the drainage capacity. This should be developed as an automatic procedure in the future. Relatively, the measures adopted in the pandemic were more appropriate since the governors and the public understood the importance and the measures had been put into practice several times in 2020.

### 4.2. Diversifying Disaster Relief Funds

**Figure 18:**
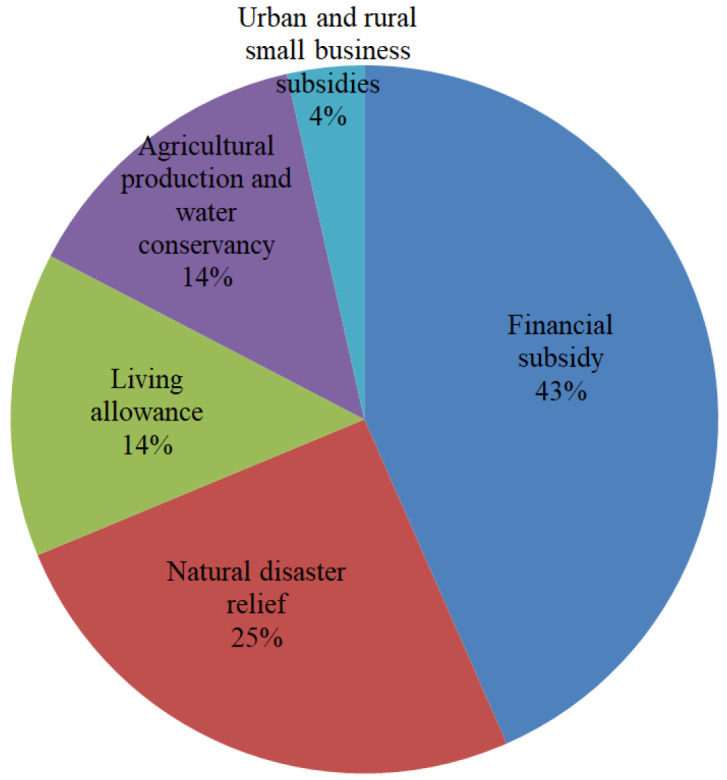
Proportions of rainstorm relief funds in five areas.

The Information Office of the provincial government held six press conferences on accelerating post-disaster reconstruction in Henan Province from August 4 to August 16. According to the released information, from July 22 to August 2, the provincial finance authority issued 11 batches of CNY 4.725 billion for rainstorm relief, which were used in five areas, in proportions as shown in Figure 18.

**Figure 19:**
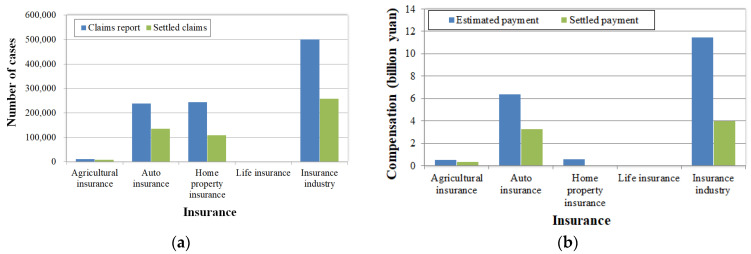
Compensation granted by insurance industry: (**a**) reported and settled cases of insurance claims; (**b**) estimated and settled claim payments.

In the face of disasters, insurance is effective for risk spreading. Up to August 10, the compensation granted by the Henan insurance industry for agricultural insurance, auto insurance, home property insurance, and life insurance was as shown in Figure 19.

A total of 501,400 claims reports were received, leading to a preliminary estimated compensation of CNY 11.449 billion, 257,600 settled cases, and a settled claim payment of CNY 4.014 billion. The rates of settled cases and claim payments in the province’s insurance industry reached 51.38% and 35.06%, respectively. For agricultural insurance, auto insurance, home property insurance, and life insurance, the rates of settled cases were 74.94%, 56.70%, 44.85%, and 42.62%, with 63.62%, 51.06%, 7.05%, and 67.64% of settled payments, respectively. Obviously, the proportions of agricultural insurance and life insurance are low and the reported and settled cases for home property insurance are high; however, the estimated and settled payments are low, which means the insurance amount was low. According to China Banking and the Insurance Regulatory Commission spokesperson’s remarks on September 7 [34], a total of 513,200 claims reports were received by the Henan insurance industry from July 17 to August 25, and the preliminary estimated compensation was CNY 12.404 billion, i.e., less than 10% of the direct loss. A total of 346,000 cases were settled, with a total settled compensation of CNY 6.885 billion. The closing rate reached 67%, where the rate for life accident insurance was 76%, for auto insurance was 86%, and for agricultural insurance was 76%.

As of August 9, the allocated financial and donated funds for disaster relief reached CNY 9.031 billion, of which an allocated CNY 5.83 billion was from the received donations of CNY 7.498 billion [12]. The relief funds were mainly from donations and financial funds, and obviously, donations in large part depend on media publicity, and cannot be regarded as a stable source of disaster relief funds. On the other hand, the insurance coverage was low, and this should be a stabilizer and contribute more as a market instrument in the future. In the face of the pandemic, the advantages of insurance and insurance-linked securities for risk decentralization and transfer should be taken into account [35].

## 5. Conclusions

In the context of climate change, we may experience more and more compound disasters, and we need to keep learning and improving countermeasures for risk prevention and mitigation. A compound disaster occurred in Henan, a central province of China, in 2021. It is time to review our responses and learn some lessons for disaster prevention in the future.

In the middle of July, Henan province suffered an extreme rainfall event, named the 7.20 Henan rainstorm. It caused 302 deaths, and 50 people were listed as missing. The direct loss was CNY 133.715 billion, as of August 9. During the process of recovery and reconstruction, local confirmed cases and asymptomatic cases of COVID-19 appeared in Zhengzhou, the capital of the province. From July 31 to August 24, 167 local cases were confirmed in this province. No mass infection appeared among the people gathering in the emergency shelter during the rainstorm, or during transfer and resettlement. We investigated the emergency response to these two events, analyzed the effects of the compound disaster and identified some lessons from this urban compound disaster. The effect of the compound disaster on provincial economic operations, the fixed-asset market, the consumer goods market, cold chain material distribution, and the logistics industry was more serious in August, but the impact on high-tech manufacturing and strategic emerging industries was positive. More enterprises in the wholesale and retail, accommodation, and catering industries were affected by the pandemic than by the rainstorm. For disaster prevention in urban areas, countermeasures as a response to the highest-level meteorological warnings, especially for rare cases, should be practical and implemented efficiently, and the sources of disaster relief funds should be diversified.

## Data Availability

The data presented in this study are openly available as references number [1,2,3,6,7,8,9,11,12,16,17,18,19,20,22,23,24,25,26,28,34].
